# Kinetic Investigation and Dissolution Behavior of Cyanide Alternative Gold Leaching Reagents

**DOI:** 10.1038/s41598-019-43383-4

**Published:** 2019-05-10

**Authors:** Alexander Birich, Srecko Stopic, Bernd Friedrich

**Affiliations:** Institute of Process Metallurgy and Metal Recycling, RWTH-Aachen University, Intzestr. 3, 52056 Aachen, Germany

**Keywords:** Geochemistry, Materials science

## Abstract

Raising social awareness and environmental specifications on cyanide application force gold industry to search for alternative leaching reagents. Therefore, researchers worldwide investigate cyanide alternatives for gold recovery since several decades. Often the research activities cannot be compared directly, since different input materials and experimental conditions are used. Over the course of this study, different promising cyanide alternative reagents were investigated in terms of their capability of pure gold dissolution at different temperatures. All experiments took place under identical conditions by using uniform samples of 99.99% gold disks, to enable a comparability. Thiosulfate as one of the most promising reagent thiosulfate according to literature revealed an insufficient leaching behavior. The gold dissolution was hindered due to the formation of a sulfide passive layer. Also in the thiourea trials, a surface precipitation took place, though gold dissolution did not stop. The halogens iodine, bromine and the well-known gold solvent aqua regia dissolved gold very fast (up to ~1,000 mg·h^−1^·cm^−2^). Methanosulfonic acid (MSA) was not capable to extract any gold. The experiments were compared with cyanide trials at identical conditions. The average dissolution rate of investigated reagents at 25 °C shows following order: aqua regia > iodine > bromine > cyanide > thiourea > thiosulfate > MSA.

## Introduction

Gold is a metal of high importance for mankind since several thousand years. Besides its applications in jewelry and as currency in former days, nowadays gold experiences a raising importance in electronics and as save funds. These fields of application are based on the excellent chemical resistance and electrical conductivity of gold. In particular its corrosion resistance is the reason for the challenging development of efficient recovery methods. Many potential reagents besides cyanide, e.g. chloride, mercury or sulfur complexes, are mostly aggressive or very toxic. Cyanide can cause severe environmental harms if handled carelessly, so through contamination of soils and groundwater. Therefore, worldwide we face environmental problems during processing of gold^1–4^. The industrial gold recovery becomes more challenging, since high grade ores are becoming rare. Gold producer are forced to use increasingly complex and low grade ores. Especially for refractory and carbonaceous preg-robbing ores cyanide leaching shows an insufficient gold recovery^2^. Besides this, also different process residues from gold winning plants and gold containing end of life products experience raising attention. These resources characteristically show strongly varying composition with elements hindering an efficient gold extraction by cyanide^5,6^. Another big disadvantage of cyanide is its low dissolution rate compared to alternative leaching reagents (lixiviants), which can be more than 10 times faster^2,7^.

The cyanide process is the dominant method for gold recovery from ores, but due to different technical and environmental issues the established cyanide process reaches its limit. In particular to overcome environmental problems and technical disadvantages, new leaching agents were increasingly researched in the past decades. Many researchers investigated various cyanide alternatives, but these activities often lack in a direct comparability, since different experimental setups, input materials and procedures were used^7–10^. This paper focusses on the investigation of the leaching behavior of different promising cyanide-alternative reagents and cyanide at identical conditions. In the following a brief description of used leaching reagents takes place^8–11^.

## Cyanide and Alternative Gold Leaching Reagents

The most commonly used cyanide salts are KCN and NaCN, which are easily soluble in water. Cyanide leaching is the dominating process for gold recovery from primary resources, due to its simplicity and high cost efficiency. Cyanide dissolves gold by forming a soluble dicyanoaurat complex (Eq. 1). The presence of oxygen is essential for the gold dissolution. To avoid the formation of volatile hydrogen cyanide HCN, the process takes place in a basic solution (pH 10). Generally, an average gold extraction yield of 50–80% can be obtained by heap or 99% by conventional leaching^2,12–14^

1 $$4{\rm{Au}}+8{{\rm{CN}}}^{-}+{{\rm{O}}}_{2}+2{{\rm{H}}}_{2}{\rm{O}}=4{\rm{Au}}{({\rm{CN}})}_{2}^{-}+4{{\rm{OH}}}^{-}$$

**Figure 1:**
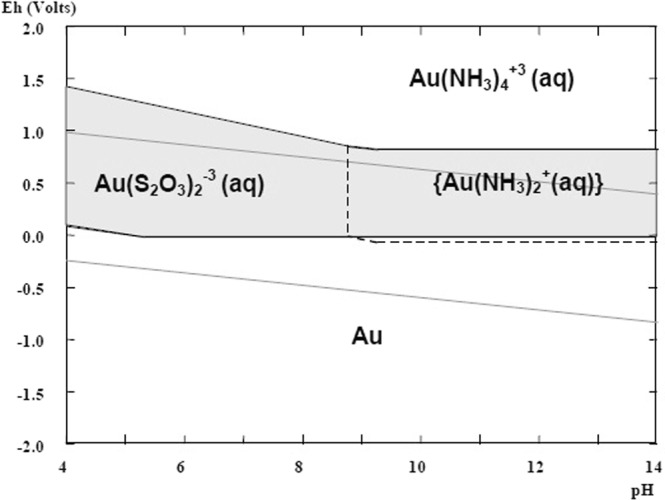
Eh-pH diagram for Au-NH_3_-S_2_O_3_^2−^ system (1 M S_2_O_3_^2−^, 10^−4^ M Au; 1 M NH^3^/NH_4_^+^)^54^.

**Figure 2:**
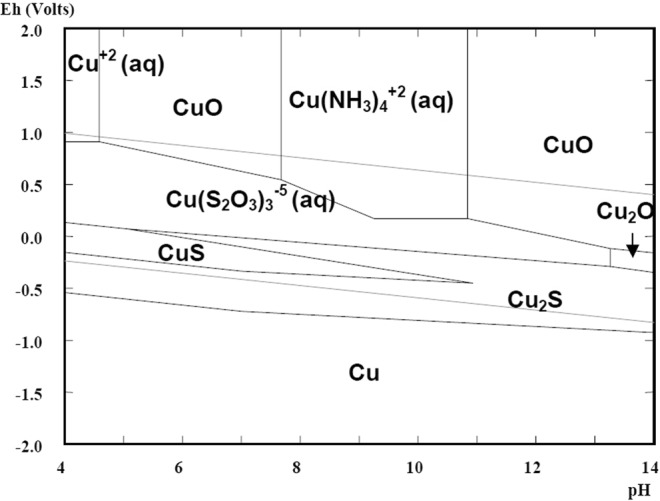
Eh-pH diagram of Cu-NH_3_-S_2_O_3_^2−^ system (0.1 M NH_3_/NH_4_^+^; 0.1 M S_2_O_3_^2−^; 5 × 10^−4^ M Cu^2+^)^54^.

Thiosulfate is the most promising non-cyanide leaching reagent and the only reagent which found industrial application on gold recovery from a highly carbonaceous preg-robbing gold ore at Barrick Gold. Besides this, the nontoxic reagent is highly discussed and there is a multitude of publications and conference papers presenting auspicious results, also for different gold containing waste types^7,15–17^. For gold extraction usually ammonium or sodium thiosulfate salts are used in an ammonia-copper leaching solution. Copper sulfate acts as a catalyst, while ammonia is a stabilizing agent for the aurothiosulfate and copper complexes. In spite of its potential environmental benefits, it is relatively slow and shows a high reagent consumption. Furthermore, the thiosulfate leaching reaction is quite complex due to several reactions taking place in parallel (oxidation of gold, redox reaction of copper, formation of copper thiosulfates and other). The stability of thiosulfate and copper complexes is strongly depending on pH-level (Figs 1 and 2). The dissolution reaction can be summarized by the following equations^17–20^.

2 $$Au+5{S}_{2}{O}_{3}^{2-}+Cu{(N{H}_{3})}_{4}^{2+}\leftrightarrow Au{({S}_{2}{O}_{3})}_{2}^{3-}+4N{H}_{3}+Cu{({S}_{2}{O}_{3})}_{3}^{5-}$$

3 $$2Cu{({S}_{2}{O}_{3})}_{3}^{5-}+8N{H}_{3}+\frac{1}{2}{O}_{2}+{H}_{2}O\leftrightarrow 2Cu{(N{H}_{3})}_{4}^{2+}+2O{H}^{-}+6{S}_{2}{O}_{3}^{-}$$

Sulfurized urea, better known as thiourea, is the next promising cyanide alternative to thiosulfate. Research activities of the past decades show considerably promising results. Thiourea (SC(NH_2_)_2_) is an organosulfur compound that forms a soluble cationic complex with gold in a sulfuric acid solution. Iron(III)sulfate is added as catalyst, facilitating the oxidation of precious metals. The dissolution reaction is described Eqs 4 and 5. Thiourea has several advantages like a fast leaching rate and a low toxicity compared to cyanide. On the other side, a high reagent consumption occurs due to the oxidation to disulfide formamidine and precipitation of sulfur. This can cause a surface passivation^5,21–25^.

4 $$Au+2SC{(N{H}_{2})}_{2}+\frac{1}{4}{O}_{2}+{H}^{+}\leftrightarrow Au{[SC{(N{H}_{2})}_{2}]}_{2}+\frac{1}{2}{H}_{2}O$$

5 $$Au+2SC{(N{H}_{2})}_{2}+F{e}^{3+}\leftrightarrow Au{[SC{(N{H}_{2})}_{2}]}_{2}^{+}+F{e}^{2+}$$

The halogens present a group of elements which show a strong tendency to form metal salts. Especially iodine, bromine and chlorine are known for their high gold dissolution rate, which is similar to aqua regia. One advantage of iodine and bromine is their high selectivity in terms of separation of precious metals from base metals. Besides their direct use as leaching reagent, a positive effect on gold recovery as additive in different leaching solutions was reported. Due to its low volatility and low hazardousness, iodine is the most convenient halogen for gold leaching. The dissolution reaction takes place in neutral or weakly alkaline solutions. Because of its rareness and high price, iodine was not used for gold recovery in industrial scale yet. Despite their advantage of a higher dissolution rate, bromine and chlorine are strongly corrosive, highly volatile and hazardous. The gold dissolution reaction is similar for all halogens (Eqs 6 to 8). The best-known gold solvent is aqua regia. In presence of the oxidant nitric acid, hydrochloric acid forms a soluble tetrachloroaurate complex^5,26–31^. In further notations aqua regia will be counted as halogenic reagent, even though strictly seen it is not. Similar to iodine and bromine, also chlorine forms a Au(I)-compound first, which afterwards reacts to A(III)-chloride with a higher stability.

6 $$2Au+{I}_{3}^{-}+{I}^{-}\leftrightarrow 2Au{I}_{2}^{-}$$

7 $${\rm{Au}}+4{{\rm{Br}}}^{-}={{\rm{AuBr}}}_{4}^{-}+3{{\rm{e}}}^{-}$$

8 $${\rm{Au}}+4{{\rm{Cl}}}^{-}={\rm{Au}}C{l}_{4}^{-}+3{{\rm{e}}}^{-}$$

Methoanosulfonic acid (MSA) belongs to the class of organosulfur compounds and is a non-oxidizing reagent. By adding a strong oxidizing agent like hydrogen peroxide, the dissolution of precious metals can be achieved. This was already reported for noble metals like silver and copper but not for gold. Following equation shows the silver dissolution of MSA. Potential advantages of MSA would be its biodegradability and the low toxicity^32,33^.

9 $$2Ag+2C{H}_{3}S{O}_{2}OH+{H}_{2}{O}_{2}\leftrightarrow 2Ag{O}_{3}SC{H}_{3}+2{H}_{2}O$$

**Figure 3:**
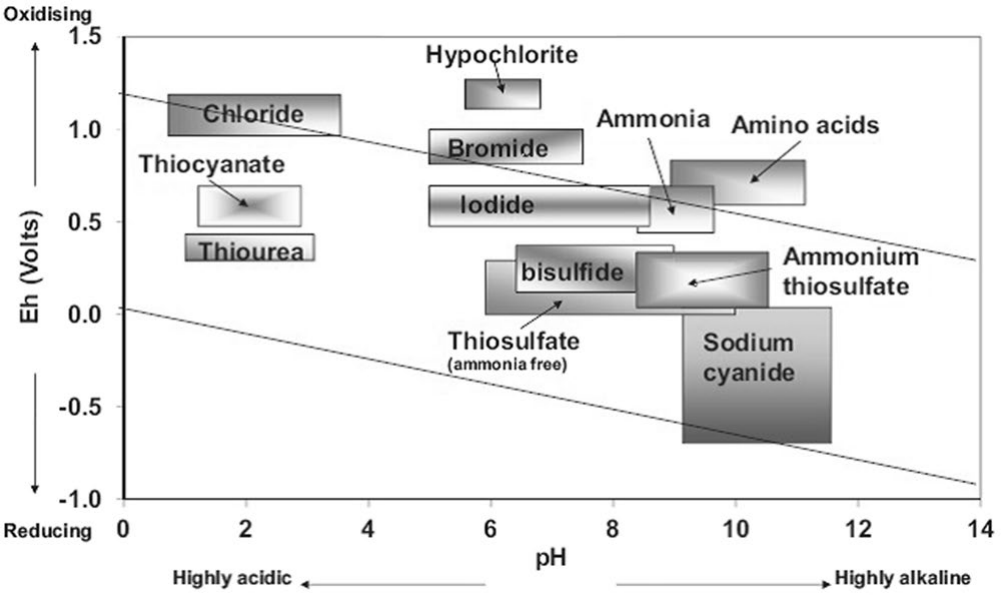
Eh-pH diagram of promising gold lixiviants and their working areas^55^.

Table 1 summarizes stability constant, standard reduction potentials and pH working area of relevant Au(I) and Au(III) complexes. An Eh-pH diagram for the different lixiviants is shown in Fig. 3.

**Table 1:** Stability constants and standard reduction potentials for relevant Au-complexes^54^.

Ligand	Complex	Log β_i_	E^0^/V	pH
S_2_O_3_^2−^	Au(S_2_O_3_)_2_^3−^	28.7	0.17	8–10
SCN^−^	Au(SCN)_2_^−^	17.1	0.66	<3
	Au(SCN)_4_^−^	43.9	0.66	
I^−^	Aul_2_^−^	18.6	0.58	5–9
	Aul_4_^−^	47.7	0.69	
Br^−^	AuBr_2_^−^	12.0	0.98	5–8
	AuBr_4_^−^	32.8	0.97	
Cl^−^	AuCl_2_^−^	9.1	1.11	<3
	AuCl_4_^−^	25.3	0.99	
CN^−^	Au(CN)_2_^−^	38.3	−0.57	>9

## Results and Discussion

### Thiosulfate

**Figure 4:**
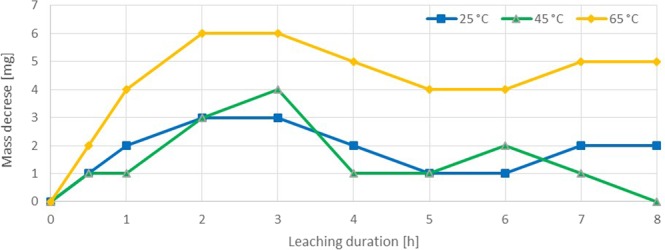
Sample dissolution vs time in thiosulfate test series (Ammonium Thiosulfate 0.3 M, Ammonia 0.3 M, Cu(II)sulfate 0.01 M).

**Figure 5:**
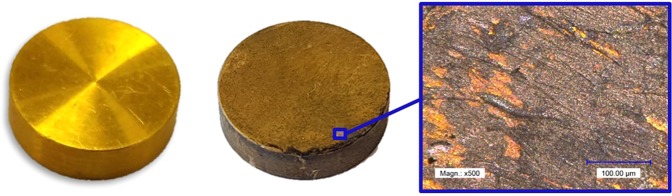
Passivation of gold surface in thiosulfate leaching experiments (from left to right: initial sample, 65 °C after 8 h leaching, optical microscopy magnification x500).

**Figure 6:**
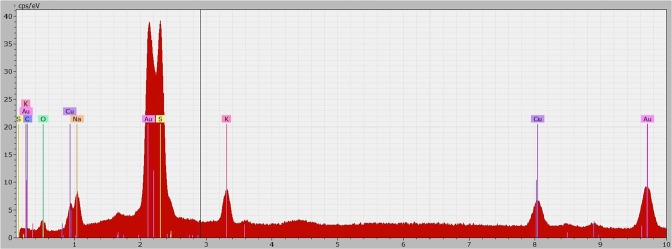
Results of EDS Analysis of passive layer on gold surface of 45 °C thiosulfate sample.

The thiosulfate leaching experiments revealed a gold dissolution rate of 0.295–1.179 mg·h^−1^·cm^−2^ at 25 °C in the first 3 hours. After 2–3 h the gold dissolution completely stopped in all experiments (Fig. 4). A subsequent mass increase was measured for all samples. The experiments were repeated, showing similar results. In all thiosulfate leaching experiments a darkening of the gold specimen surface was observed. At higher solution temperatures the surface became darker than in the 25 °C trial. This effect was clearly visible especially for the trials at 65 °C (Fig. 5). The color indicates the presence of sulfidic copper or gold compounds^34,35^, which was confirmed by an EDS analysis. On different positions, there was a significant peak for Cu and S, as shown in Fig. 6. The surface layer was insoluble, as 48 and 72 h experiments showed. The elements Na and K, which are displayed in the EDS diagram, origin from the used reagents (purity >98%) and de-ionized water. Since these elements are present in most systems, no negative effect is expected or was reported in literature. The thiosulfate leaching of the Au80Ag20 alloy did not improve dissolution behavior.

The occurrence of a surface layer can be explained by the formation of a passive layer. The stability of the ammonia thiosulfate system is strongly dependent on the concentration of copper amines, oxygen and the ammonia. The atmospheric leaching setup enables a permanent supply of oxygen, which not only facilitates the gold oxidation but also increases thiosulfate decomposition at the same time. Polythionates or sulfate compounds are formed by oxidation of thiosulfate. These products are adsorbed on the gold surface and hinder further the dissolution reaction. Furthermore, the permanent ammonia evaporation causes a drop of the pH level, in particular at higher temperatures. This effect strongly influences the stability of soluble copper complexes and may lead to the precipitation of copper sulfides or oxides (Fig. 2). In particular at elevated temperatures the formation of insoluble compounds is facilitated due to the reagent instability and reduced reaction energy^36–40^. The precipitate leads to an increasing sample mass and a slowing down or stopping of the dissolution reaction.

### Thiourea

**Figure 7:**
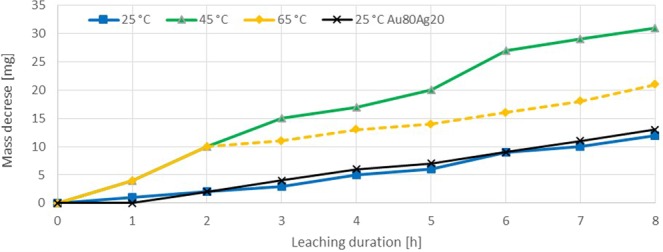
Sample dissolution vs time in thiourea test series (Thiourea 0.26 M, 0.02 M Fe(III)sulfate, 0.15 M H_2_SO_4_).

The thiourea leaching experiments show a continuous and constant gold extraction over the time in all experiments (Fig. 7). A significant effect of leaching temperature was observed in these experiments. The mean dissolution rate of the trial at 45 and 65 °C were up to two times faster than that of the 25 °C trial (25 °C: 0.516 mg·h^−1^·cm^−2^; 45 °C: 1.142 mg·h^−1^·cm^−2^; 65 °C: 0.774 mg·h^−1^·cm^−2^). At 65 °C a conspicuous decrease in leaching rate took place after 2 hours. Unlike trials at lower temperatures, at 65 °C a strong evaporation took place causing a drop of pH from 0.9 to 0.6 and lower. To stabilize the pH value, deionized water was added after 3 and 6 hours, enabling to maintain and temporarily increase the dissolution rate. The addition of de-ionized water during the experimental procedure causes a condition, which differs from other experiments. This precludes the comparability to lower leaching temperatures or other reagents. Therefore, the dissolution course after 3 hours have to be considered with reservation. For the 25 and 45 °C trials the pH value remained constant between 0.8–1.1.

In all thiourea leaching experiments the specimens became slightly darker with time, but unlike the thiosulfate experiments the dissolution reaction did not slow down or stop. The change in color can be explained with the formation of a passive layer, which origins from the oxidation of thiourea with oxygen and due to a redox-reaction with the catalyst iron. This mechanism leads to adsorption of polysulfides and sulfate ions on the gold surface. By controlling pH and oxygen concentration, this effect can be reduced. The high thiourea concentration in conducted trials facilitate the reagent decomposition. Unlike the thiosulfate system, the passive layer is soluble and enables further gold leaching^41–44^.

Besides pure gold samples, also a gold-silver specimen was used in one trial to determine the influence of the alloying element silver in thiourea leaching systems. As can be seen in Fig. 7, silver does not have distinct effect on the sample dissolution. The rate determining step is the diffusion of Au- or Ag-thiourea complexes though the passive layer. Since the diffusion constants are similar for both complexes, the dissolution of the gold alloy was not substantially faster than for pure gold. In general, silver was found to be more sensitive in Fe^2+^/Fe^3+^ catalyzed thiourea leaching systems. The dissolution of pure gold was observed to be 2–4 times faster than for pure silver^24,25,41,45,46^.

### Iodine

**Figure 8:**
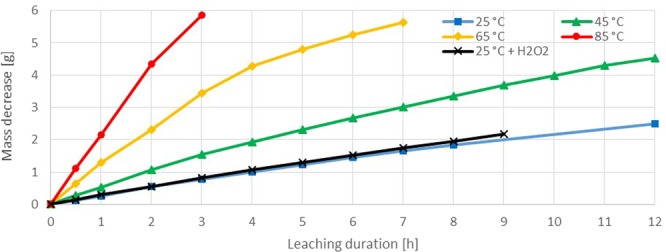
Sample dissolution vs time in iodine test series (iodine 0.08 M, potassium iodide 0.80 M).

Iodine experiments revealed a fast gold dissolution reaction (Fig. 8). At 25 °C 0.26 g/h (80 mg·h^−1^·cm^−2^) and for 85 °C even 2.16 g/h (702 mg·h^−1^·cm^−2^) of gold were leached at the beginning. The slowing down of the dissolved gold mass per hour can be explained with the decreasing surface and the decreasing free iodine-ions in solution. So the increasing diffusion zone of iodine to the gold surface is limiting the leaching reaction with the time. Just the trial at 85 °C shows a nearly lineal trend in mass decrease, but this is most probably results from the low quantity of measurement points. The experiments showed a strong temperature influence on the gold-iodine reaction due to increasing diffusion and/or decreasing activation energy. In one experiment at 25 °C hydrogen peroxide was added to raise the oxidative property of the solution. The oxidant increased the reaction kinetics slightly in the first hours. After 8 hours the dissolution reaction is more than 25% faster (first hour 10% deviation: 80 and 87 mg·h^−1^·cm^−2^; after 8 hours 25% deviation: 62 and 78 mg·h^−1^·cm^−2^). This observations reveals that another reason for the decreasing reaction kinetics may be the consumption of dissolved oxygen and herewith lower oxidative solution properties. The solubility of gases like oxygen in solution is negatively correlated to the temperature and concentration of other dissolved compounds, like metal salts^47,48^. Since with progression of time the metal ion concentration increases, a de-aeration of oxygen takes place.

**Figure 9:**
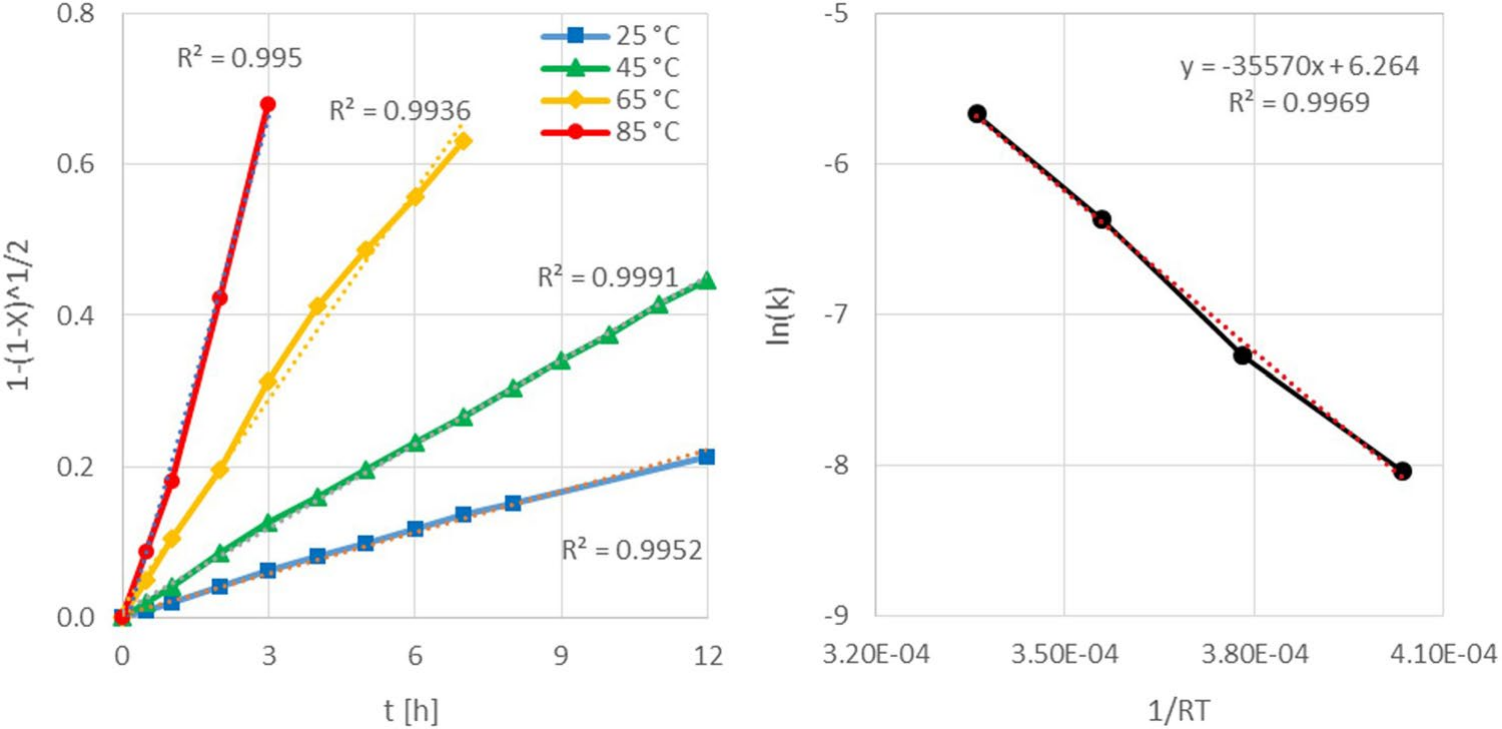
Adaption of shrinking core model on cylindric object.

Kinetical calculations were conducted to determine the dissolution model and activation energy of the gold leaching with iodine. After comparing different models, it was found that the shrinking core model for a cylindrical geometry suits to the experimental data (Fig. 9)^49,50^. A reaction controlled system was determined for gold-iodine leaching. The activation energy was found to be 35.57 kJ/mol, which fits in the range of calculations from other researchers^51,52^.

### Bromine

**Figure 10:**
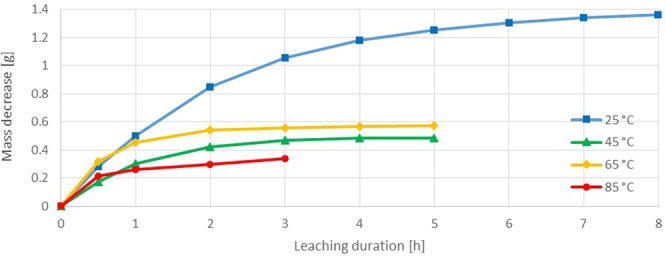
Sample dissolution vs time in bromine test series (bromine 0.20 M, potassium bromide 0.67 M).

Due to their chemical similarity but higher corrosiveness, it is expected that bromine will have better leaching characteristics as iodine. Especially at the beginning of the 25 °C trial, the dissolution rate of bromine was much higher than for all other reagents (154.042 mg·h^−1^·cm^−2^) (Fig. 10). The strong evaporation of bromine already in the first hours lead to a decreasing leaching rate. This results in an average leaching rate of just 57.765 mg·h^−1^·cm^−2^. With increasing temperature the bromine evaporation intensified, so that the 85 °C trial showed the lowest gold dissolution. Since the significant evaporation of bromine does not enable a representative observation of its gold leaching properties, the experiments at higher temperatures than 25 °C were stopped already after 5 or 3 hours. At 65 °C a slightly stronger mass decrease than in the 45 °C trial was observed, which can be explained with increased leaching kinetics at higher temperatures. The increased leaching kinetics at this temperature change were also observed in the iodine experiments (Fig. 8). To prove that the dissolution reaction slows down due to a reagent loss by evaporation, in the trials at 45 and 65 °C 5.14 ml bromine was added after 5 hours (not displayed). This led to an eminent improvement of the gold dissolution from 6.36 and 2.96 mg·h^−1^·cm^−2^ to 298.05 and 262.72 mg·h^−1^·cm^−2^.

### Aqua Regia, MSA and Cyanide

**Figure 11:**
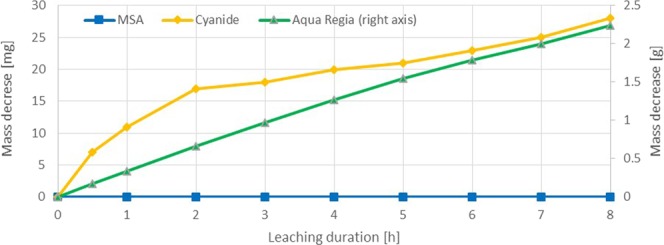
Sample dissolution vs time for MSA (0.5 M), KCN (0.14 M) and aqua region (right axis) at 25 °C.

Leaching experiments with aqua regia revealed that the reagent has the highest average dissolution rate of all reagents at 25 °C (84.239 mg·h^−1^·cm^−2^). The trials showed a very similar leaching behavior to the iodine and bromine experiments. At high chlorine concentrations in solution, the mass transport of chloride ions to the gold surface by diffusion is negligible^2^. This is the case for the present system. Therefore the dissolution rate determining step is the gold-chlorine reaction, which is much faster than for other reagents, in particular non-halogens (Fig. 11). This is approved by the linear mass decrease of the gold specimen. Although it is expected that higher temperatures will enable accelerated leaching conditions, as observed for iodine, no trials above 25 °C were conducted. Similar to bromine, also hydrochloric acid has a low boiling point (45 °C), which would lead to a reagent loss and a slowing down of gold extraction.

**Figure 12:**
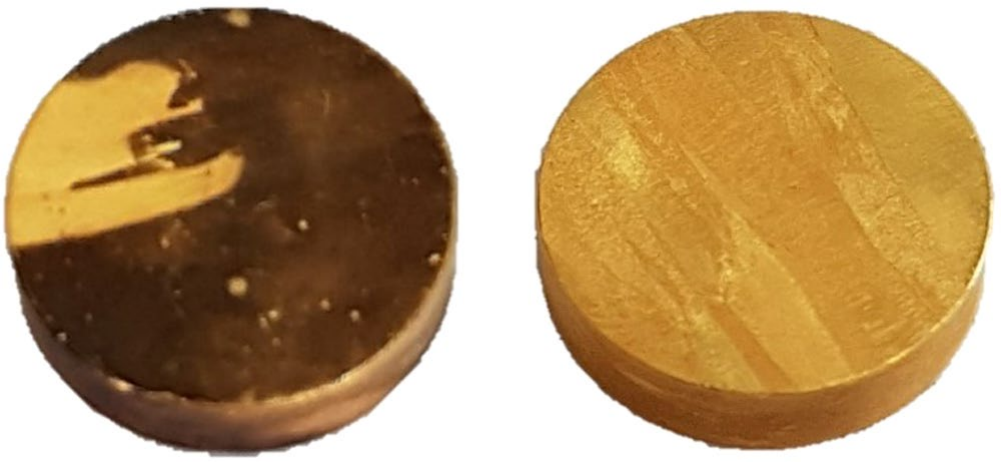
Gold specimen in cyanide test (0.14 M KCN) after 3 h (left) and after 6 h (right picture).

The average dissolution rate of cyanide leaching (1.032 mg·h^−1^·cm^−2^) fits the expectation from literature^2^. Also in the cyanide system a change in color of the specimen was observed. On the surface a dark layer was formed in the first hours, which slows down dissolution rate already in the first two hours. Unlike the thiosulfate experiments, this coating is nor compact neither stable. Already by the flow stream caused by stirring, the coating was removed and dissolved completely (Fig. 12). The occurrence of this dark coating is based on the formation of AuCN, which is formed in an intermediate reaction. The AuCN reacts with CN^−^ and form soluble ${\rm{Au}}{({\rm{CN}})}_{2}^{-}$. Under certain conditions, like in systems with high cyanide and oxygen concentrations, the AuCN formation is faster than its evacuation, causing a precipitation on the gold surface due to exceeding the local solubility limit^53^. To prove this explaination, three cyanide leaching experiments with a lower KCN concentration of 0.07, 0.004 and 0.002 M KCN were conducted. The lower cyanide concentration provides a reduced AuCN formation rate. For 0.07 M cyanide just a very weak coating was observed, for 0.04 and 0.02 no coating was visible anymore. By reducing the KCN concentration also the dissolution rate dropped significantly to around 60, 40 and 20% respectively, compared to the initial leaching test (0.14 M KCN).

The organic and non-oxidizing reagent MSA was not capable to extract any gold over 8 hours. Neither in experiments with addition of hydrogen peroxide nor the use of a gold-silver specimen or increased leaching time enabled a measureable mass decrease of the gold specimen. Unlike for silver, the oxidizing conditions do not suffice the gold leaching^32,33^.

## Conclusions

The conducted experiments revealed important information about the leaching behavior of different gold leaching reagents at defined temperatures under identical conditions. Most experiments indicate a significant influence of oxygen on the dissolution behavior of investigated gold leaching reagents. The oxygen activity in solution is varying over time depending on the metal salt concentration and temperature. Another important observation is the negative influence of temperature on volatile leaching reagents or on for different reagents.

- Thiosulfate as most promising cyanide alternative lixiviant was not capable to dissolve pure or silver-alloyed gold samples. The thiosulfate leaching experiments caused the formation of a stable passive layer after 2–3 hours, which led to a mass increase (0.3 M thiosulfate and ammonia, 0.01 copper sulfate). This compact coating hindered any further gold dissolution. An EDS analysis revealed the composition of the passive layer: it consists of an Cu-S-complex.
- Thiourea showed a continuous gold dissolution over time (0.26 M thiourea, 0.02 M iron sulfate, 0.15 M sulfuric acid). The average dissolution rate of thiourea at 25 °C was lower than cyanide (0,516 and 1,032 mg·h^−1^·cm^−2^). Silver as an alloying element did not affect the sample dissolution. At temperature above 45 °C, the thiourea solution evaporated non-equimolar, causing a drop in pH and herewith slowing down leaching kinetics.
- The leaching experiments with iodine, bromine and aqua regia showed the fastest and continuous gold dissolution over time. A significant influence of temperature on gold leaching was observed in all experiments.
  - Iodine is capable to extract gold a very fast (average dissolution rate of 65.679 mg·h^−1^·cm^−2^ at 25 °C). At higher temperature the dissolution rate increased up to 806.161 mg·h^−1^·cm^−2^. It was found that the iodine dissolution of gold is reaction controlled. An activation energy of 35 kJ was determined for the iodine leaching reaction.
  - Bromine leaching was faster than any other reagent in the first 3 hours at 25 °C. But with time the dissolution rate dropped due to the evaporation of bromine. The lixiviant has a high vapor pressure of 20 kPa^35^, causing a strong reagent loss into the gas phase. Leaching in closed pressure reactor would be favorable for bromine to avoid the reagent evaporation. For iodine and bromine a significant amendment of the leaching kinetics was observed at a temperature increase from 45 to 65 °C.
  - Aqua regia showed a higher average dissolution rate over eight hours than iodine, bromine or any other reagents at 25 °C.
- MSA is not capable to extract gold, neither by addition of the oxidizing agent hydrogen peroxide.

The extremely fast gold dissolution rate of iodine and bromine solutions can be compared with aqua regia, which is already used in small scale gold recovery processes. In contrast with aqua regia these reagents are more stable, less corrosive and have a lower toxicity, thus are promising reagents for industrial gold recovery. Nevertheless, due to high reagents prices of iodide and bromide a loss-free process has to be developed to enable an economic process. Thiosulfate is reported to be the most promising cyanide alternative, but shows some significant drawbacks which are based on the low reagent stability and may cause a surface passivation. This may be the reason for the limited industrial application of thiosulfate. With a quite similar dissolution rate as cyanide, the experiments did not show an advantage of thiourea for the gold recovery from gold ores.

## Materials and Methods

**Figure 13:**
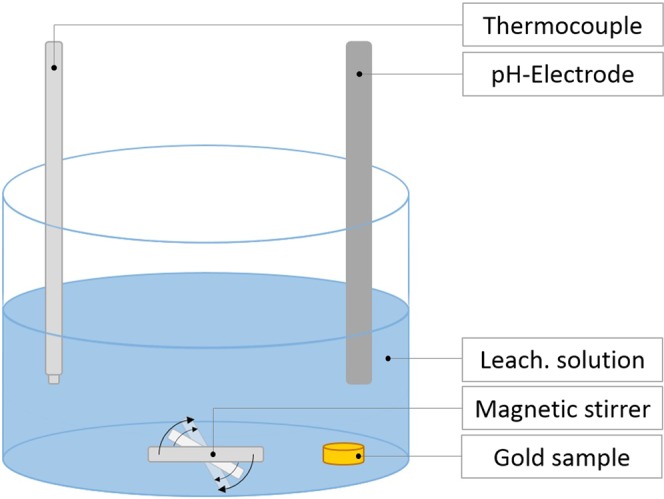
Schematic diagram of the reaction vessel.

All leaching experiments took place in an open to atmosphere beaker (1 L) on a stirred heating plate (SLR, SI Analtics) by controlling the solution temperature with a PTFE coated thermocouple. The pH value was measured with a pH/mV measuring device (ph1970i, WTW). A schematic diagram of the experimental setup is shown in Fig. 13. The investigated reagent grade lixiviants were acquired from AlfaAesar and SigmaAldrich.

As material of investigation, identical cylindrical gold samples were provided by Varinor SA. The samples showed following properties: a gold purity of 99.99 wt%; a surface area of 3.393 cm² (diameter 12.0 mm, height 3.0 mm) and a mass of 6.526 g +/− 0.005. For selected experiments a gold disk containing 20 wt% silver was used to investigate the leaching behavior of a common gold alloy. The Ag20Au-disks were produced at the IME and are identical in geometry compared to the pure gold specimens. The initial mass of the Ag20Au disks is lower (5.621 g +/− 0.012). For sample weighting a special accuracy weighing device was used (XS603S DeltaRange, Mettler Toledo). Surface analysis and microscopy scanning electron microscopy (SEM) and electron dispersive spectroscopy (EDS) took place with a FEI SEM (Quanta-650F SEM, Thermo Fisher Scientific).

All experiments were conducted at identical conditions. A solution volume of 500 ml (solid liquid ratio of ~1/80) was adapted at an agitation speed of 250 rpm. The reagent grade lixiviants were added in excess to enables a permanent reagent excess over the experimental time. After reaching aimed temperature the experimental time started. The gold samples were always put on the same position to enable comparable stirring conditions. The samples were measured in an hourly time interval to determine mass and volume decrease over time. The measurement consisted of taking out the sample from leaching solution, drying with compressed air, measurement of weight and geometry and putting it back. This procedure took 1 min on average, which is not considered in the total leaching time (i.e. there is a negligible deviation between of displayed and real leaching time of ~1.7%). To avoid a slowing down of the dissolution reaction due to a lack of free reagents, all lixiviants were added in a stoichiometric ratio of 3–5 to 1 mole gold. Table 2 gives an overview of conducted experiments and their respective parameters.

**Table 2:** Conducted leaching experiments and respective parameters.

Test	Solution composition (Σ 500 ml)	T (°C)
1–3	Ammonium Thiosulfate 0.3 M, NH_3_ 0.3 M, CuSO_4_ 0.01 M	25, 45, 65
4	Amm. Thiosulfate 0.3 M, NH_3_ 0.3 M, CuSO_4_ 0.01 M, Au80Ag20	25
5–8	Thiourea 0.263 M, Fe_2_(SO_4_)_3_ 0.02 M, H_2_SO_4_ 0.15 M	25, 45, 65
9	Thiourea 0.263 M, Fe_2_(SO_4_)_3_ 0.02 M, H_2_SO_4_ 0.15 M, Au80Ag20	25
10–13	Iodine 0.08 M, KI 0.80 M	25, 45, 65, 85
14	Iodine 0.08 M, KI 0.80 M, 7.5 ml H_2_O_2_	25
15–18	Bromine 0.20 M, KBr 0.67 M	25, 45, 65, 85
19	Methane sulfonic acid 0.5 M	25
20	Methane sulfonic acid 0.5 M, H_2_O_2_ 1 M	25
21	Methane sulfonic acid 0.5 M with Au80Ag20	25
22	Aqua Regia (HCl:HNO_3_ = 3:1)	25
23	Potassium cyanide 0.14 M	25
24	Potassium cyanide 0.07 M	25

Main parameter of investigation is the temperature, so leaching experiments took place at 25, 45, 65 and 85 °C. In case of thiosulfate and thiourea, leaching at 85 °C did not take place, since a non-equimolecular evaporation took place causing a significant change in pH, which was already observed at 65 °C. For both reagents a defined pH interval is necessary to enable suitable leaching conditions. Since these two reagents are the most promising cyanide alternatives for gold ores, a gold-silver alloy was used to see the effect of alloying elements onto the dissolution reaction.

Besides the four promising cyanide alternatives, also MSA was tested at 25 and 45 °C with and without the addition of hydrogen peroxide. To enable a comparability of the alternative reagents, the established lixiviants cyanide and aqua regia were also tested at 25 °C and identical leaching conditions.
